# Crystal structure, Hirshfeld surface analysis and PIXEL calculations of a 1:1 epimeric mixture of 3-[(4-nitro­benzyl­idene)amino]-2(*R,S*)-(4-nitro­phenyl)-5(*S*)-(propan-2-yl)imidazolidin-4-one

**DOI:** 10.1107/S2056989019013938

**Published:** 2019-10-29

**Authors:** Ligia R. Gomes, John Nicolson Low, James L. Wardell, Marcus V. N. de Souza, Cristiane F. da Costa

**Affiliations:** a REQUIMTE, Departamento de Química e Bioquímica, Faculdade de Ciências da Universidade do Porto, Rua do Campo Alegre, 687, P-4169-007 Porto, Portugal; b FP-ENAS-Faculdade de Ciências de Saúde, Escola Superior de Saúde da UFP, Universidade Fernando Pessoa, Rua Carlos da Maia, 296, P-4200-150 Porto, Portugal; c Department of Chemistry, University of Aberdeen, Meston Walk, Old Aberdeen AB24 3UE, Scotland; d Instituto de Tecnologia em Fármacos–Farmanguinhos, Fundaçâo Oswaldo Cruz, 21041-250 Rio de Janeiro, RJ, Brazil

**Keywords:** crystal structure, Hirshfeld surface analysis, PIXEL calculations, epimeric mixture

## Abstract

A 1:1 epimeric mixture of 3-[(4-nitro­benzyl­idene)amino]-2(*R,S*)-(4-nitro­phen­yl)-5(*S*)-(propan-2-yl)imidazolidin-4-one, C_19_H_19_N_5_O_5_, was isolated from a reaction mixture of 2(*S*)-amino-3-methyl-1-oxo­butane­hydrazine and 4-nitro­benz­alde­hyde in ethanol. The product was derived from an initial reaction of 2(*S*)-amino-3-methyl-1-oxo­butane­hydrazine at its hydrazine group to provide a 4-nitro­benzyl­idene derivative, followed by a cyclization reaction with another mol­ecule of 4-nitro­benzaldehyde to form the chiral five-membered imidazolidin-4-one ring. The formation of the five-membered imidazolidin-4-one ring occurred with retention of the configuration at the 5-position, but with racemization at the 2-position. In the crystal, N—H⋯O(nitro) hydrogen bonds, weak C—H⋯O(carbon­yl) and C—H⋯O(nitro) hydrogen bonds, as well as C—H⋯π, N—H⋯π and π–π inter­actions, are present. These combine to generate a three-dimensional array. Hirshfeld surface analysis and PIXEL calculations are also reported.

## Abstract 2

A 1:1 epimeric mixture of 2(*R,S*)-(4-nitro­phen­yl)-3-[(4-nitro­benzyl­idene)amino]-5(*S*)-(propan-2-yl)imidazolidin-4-one was derived from an initial reaction of 2(*S*)-amino-3-methyl-1-oxo­butane­hydrazine at its hydrazine moiety to provide a 4-nitro­benzyl­idine derivative, followed by a cyclization reaction with another mol­ecule of 4-nitro­benzaldehyde to form the chiral five-membered imidazolidin-4-one ring.

## Chemical context   

Imidazolidin-4-ones have been widely studied (Blackmore & Thompson, 2011 ▸) due to their wide range of uses, for example, as chiral ligands in catalysis (Lin *et al.*, 2013 ▸; Mondini *et al.*, 2013 ▸; Seebach *et al.*, 2008 ▸; Puglisi *et al.*, 2013 ▸) and for their biological activities (Elrod & Worley, 1999 ▸; Gomes *et al.*, 2004 ▸; Guerra *et al.*, 2011 ▸; Barrow *et al.*, 2007 ▸). As a consequence of their utility, there are a number of well-established synthetic routes, in particular those involving chiral synthesis (Blackmore & Thompson, 2011 ▸; Eyilcim *et al.*, 2018 ▸; Li *et al.*, 2004 ▸; Vale *et al.*, 2008 ▸, 2009 ▸; Catalano *et al.*, 2011 ▸; Xu *et al.*, 2010 ▸). As part of our studies on nitro­gen-containing heterocyclic com­pounds, we report the crystal structure, Hirshfeld surface analysis and PIXEL calculations of a 1:1 epimeric mixture of 3-[(4-nitro­benzyl­idene)amino]-2(*R,S*)-(4-nitro­phen­yl)-5(*S*)-(pro­pan-2-yl)imidazolidin-4-one, **1**.

## Structural commentary   

**Figure 1:**
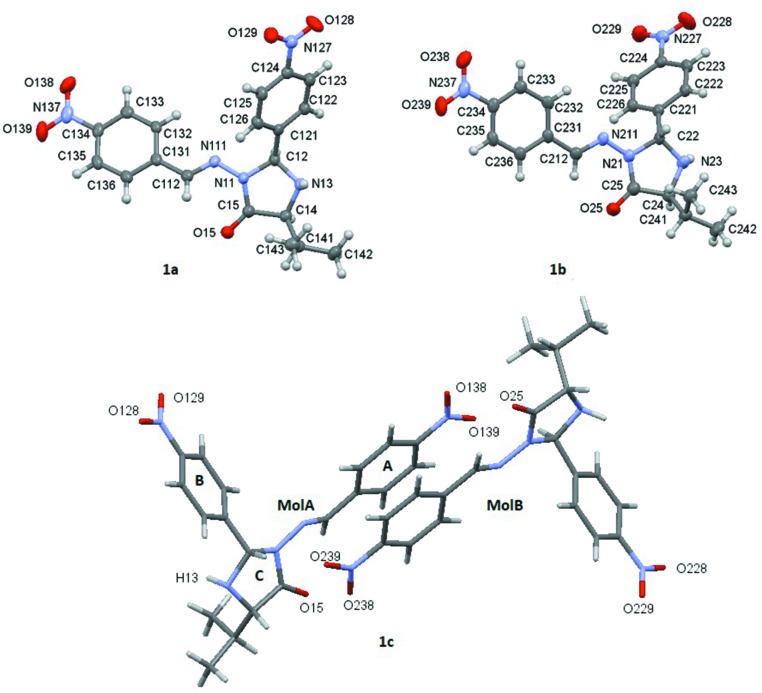
Compound **1**, showing the mol­ecular structures and numbering schemes for (*a*) **MolA** and (*b*) **MolB**. Displacement ellipsoids are drawn at the 50% probability level. (*c*) The asymmetric unit containing **MolA** and **MolB**, with rings designated as *A*, *B* and *C*.

The title com­pound, **1**, contains one mol­ecule each of the epimers in the asymmetric unit. The 3-[(4-nitro­benzyl­idene)amino]-2(*S*)-(4-nitro­phen­yl)-5(*S*)-(propan-2-yl)imidazolidin-4-one stereoisomer is termed **MolA** and the 3-[(4-nitro­benzyl­idene)amino]-2(*R*)-(4-nitro­phen­yl)-5(*S*)-(propan-2-yl)imidazolidin-4-one stereoisomer is termed **MolB** (see Figs. 1 ▸ *a* and 1*b*). In **MolA**, the configurations at atoms C12 and C14 are *S*. In **MolB**, the configurations at atoms C12 and C14 are *R* and *S*, respectively (Fig. 1 ▸). The asymmetric unit is shown in Fig. 1 ▸(*c*).

In both mol­ecules, the imidazoline rings are puckered, the puckers in each case being a twist at C12—N13 and C22—N23 in **MolA** and **MolB**, respectively. In the case of **MolA**, the Cremer & Pople puckering parameters (Cremer & Pople, 1975 ▸) are *Q*(2) of 0.287 (2)Å and φ(2) of 54.7 (5)° for reference bond N11—C12; for **MolB**, *Q*(2) is 0.103 (3)Å and φ(2) is 230.3 (15)° for reference bond N21—C22. In **MolA**, the dihedral angles between the mean planes of the imidazoline ring and the benzene ring (pivot atom C121) is 45.83 (18)°, between the imidazoline ring and the benzene ring (pivot atom C131) is 28.04 (12)° and between the two benzene rings is 69.86 (11)°. In **MolB**, the dihedral angles between the mean planes of the imidazoline ring and the benzene ring (pivot atom C221) is 59.83 (13)°, between the imidazoline ring and the benzene ring (pivot atom C131) is 6.86 (13)° and between the two benzene rings is 66.38 (11).

## Supra­molecular features   

### Inter­molecular inter­actions and contacts   

As seen, each of the mol­ecules of the asymmetric unit (Fig. 1 ▸ *c*) has two nitro groups, whose O atoms can act as acceptors for hydrogen bonding, and three rings that are able to participate in π–π stacking. Fig. 1 ▸(*c*) shows the two mol­ecules labelled for the nitro O atom and the oxo atoms (O15 and O25), as well as the identification of ring *A* (benzene rings with pivot atoms C131 and C231), *B* (benzene rings with pivot atoms C121 and C221) and *C* (imidazoline rings).

**Figure 2:**
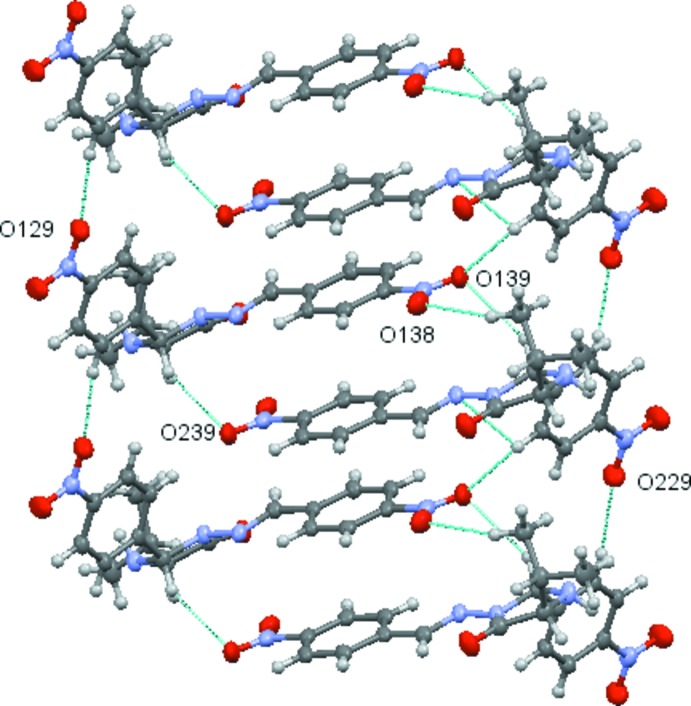
Dimers of π–π stacked **MolA** and **MolB**, which com­prise the asymmetric unit, further linked by π–π inter­actions extending the chain by unit translation along the *a* axis. The π–π inter­actions are augmented by C—H⋯O hydrogen bonds.

**Figure 3:**
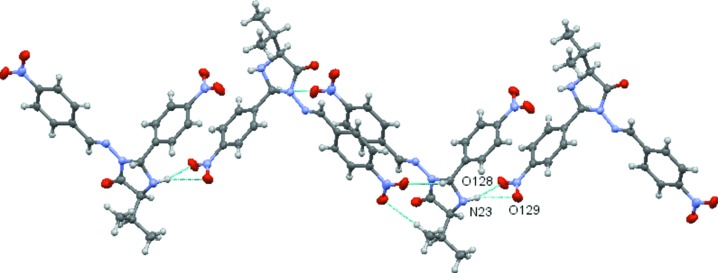
Part of a chain of mol­ecules linked by N23⋯·O128/129 hydrogen bonds connect the asymmetric unit dimers into a chain. Only the atoms in the N23⋯O128/O129 three-centred hydrogen bond are labelled for clarity.

A *PLATON* analysis (Spek, 2009 ▸) indicates the possibility in **1** of N—H⋯O(nitro), C—H⋯O(nitro) and C—H⋯O(oxo) hydrogen bonds, and C—H⋯π, N—O⋯π and π–π inter­molecular inter­actions. All details of the hydrogen bonding (mol­ecular contacts) and π–π stacking are given in Tables 1 ▸ and 2 ▸, respectively. Noticeable among these is the three-centred hydrogen bond between N23 in **MolB** and the nitro-group atoms O128/O129 in **MolA** (symmetry code: *x* + 1, *y* − 1, *z* + 1), which generate chains running parallel to the [1
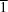
1] direction. Within the chosen asymmetric unit (see Fig. 1 ▸ *c*), the benzene rings with pivot atoms C131 and C231 are π–π stacked, forming a dimer. This stacking is supplemented by the C22—H22⋯O139, C243—H24*D*⋯O138 and C12—H12⋯O239 weak hydrogen bonds. Details are given in Tables 1 ▸ and 2 ▸. Such π–π-linked dimers are linked by further π–π inter­actions, forming a π–π stacked column, which extends along the *a* axis by unit translation (see Table 2 ▸). The C122—H122⋯O129 and C224—H224⋯O229 weak hydrogen bonds 
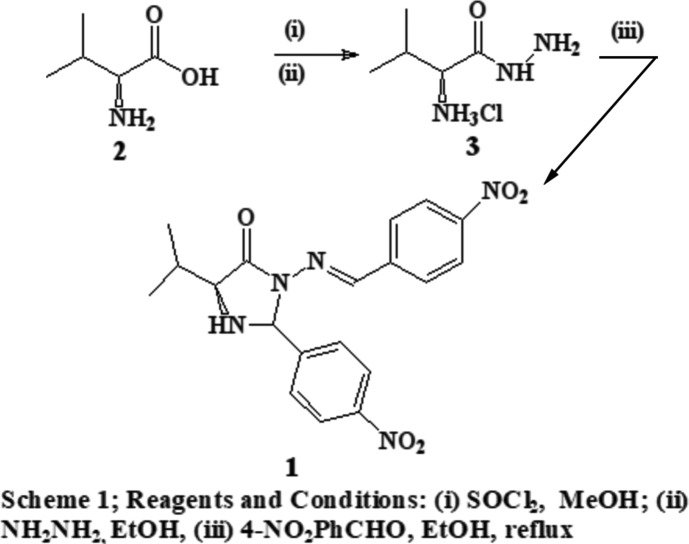
supplement the inter­dimer π–π stacking (Fig. 2 ▸). These π–π-stacked dimers are also linked by the N23—H23⋯O128/O129 hydrogen bond described above; this inter­action creates chains, which propagate parallel to the [1
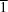
1] direction (Fig. 3 ▸, see Table 1 ▸ for details). The C112—H112⋯ O15 and C212—H21⋯O25 are possible intra­molecular hydrogen bonds. The C133—H133⋯O15(*x*, *y* − 1, *z*) and C233—H233⋯O25(*x*, *y* − 1, *z*) hydrogen bonds, found by *PLATON*, separately create *C*(9) chains that propagate in the direction of the *b* axis. There is one inter­molecular C—H⋯π inter­action involving C143—H14*A*⋯*Cg*2(*x*, *y* + 1, *z*) [*Cg*2 is the centroid of the benzene ring with pivot atom C121(*x*, *y* + 1, *z*)], with an H⋯*Cg*2 distance of 2.95°, an angle at H of 128° and a C143⋯*Cg*2 distance of 3.638 (3)°.

### Hirshfeld surface and qu­anti­tative analyses of inter­molecular inter­actions   

**Figure 4:**
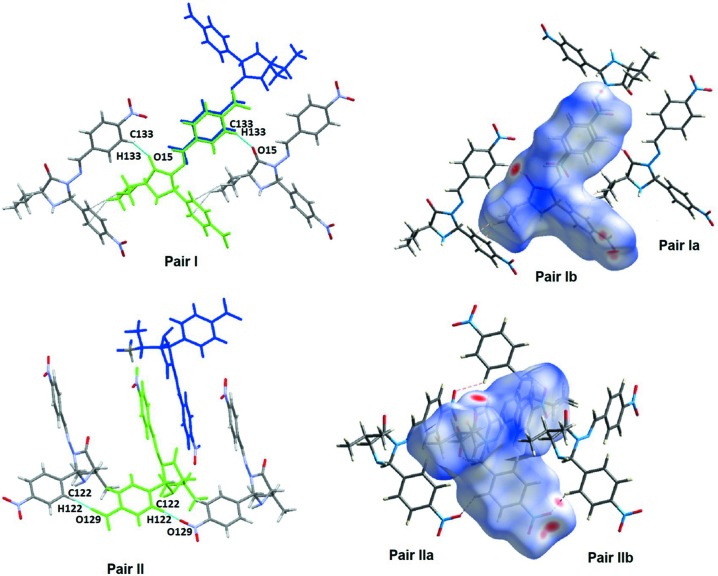
Inter­actions connecting mol­ecule pairs I and II, and a view of the Hirsfeld surface.

Hirshfeld surfaces (Spackman & Jayatilaka, 2009 ▸) and two-dimensional fingerprint (FP) plots (Spackman & McKinnon, 2002 ▸) provide com­plementary information concerning the inter­molecular inter­actions deduced from the *PLATON* analysis. The Hirshfeld analysis, generated using *CrystalExplorer* (Version 3.1; Wolff *et al.*, 2012 ▸) and mapped over *d*_norm_ (ranging from −0.329 to 1.708), indicated red areas related to specific inter­molecular short contacts (see Figs. 4 ▸–7 ▸ ▸ ▸).

Briefly, the Hirshfeld surface analysis revealed that in **MolA** all the O atoms participate in hydrogen bonding, but in **MolB** only three do, the exception being O238 in ring *A*. A summary of these inter­actions is made in Table 3 ▸. Carbonyl atoms O15 or O25 of heterocyclic ring *C* and nitro atoms O129 or O229 of ring *B* are involved in hydrogen bonding between two similar mol­ecules, *i.e.* **MolA**⋯**MolA** or **MolB**⋯**MolB**. Those pairs inter­act in a similar way. All the nitro-group O atoms of **MolA** (O128, O129, O138 and O139) act as acceptors for H atoms of **MolB**.

PIXEL energy calculations, as implemented in *PIXEL3.1* (Gavezzotti, 2003 ▸, 2008 ▸), were run in order to calculate the total stabilization energy of the crystal packing, *E*_tot_, distributed as Coulombic, *E*_Coul_, polarization, *E*_pol_, dispersion, *E*_disp_, and repulsion, *E*_rep_, terms. Partial analysis of the PIXEL calculations have been made and the results obtained were used to identify pairs of mol­ecules within the crystal network that most contribute to the total energy of the packing.

The com­pound crystallized with two mol­ecules (**MolA** and **MolB**) in the asymmetric unit and each has five O atoms that may be involved in the formation of hydrogen bonds, which are labelled in Fig. 1 ▸(*c*). In short, each mol­ecule has two 4-NO_2_-phenyl substituents, one substituent connected to the imine C atom, ring *A* (pivot atoms C131 and C231 in **MolA** and **MolB**, respectively), and the other to the imidazoline ring, ring *C* (pivot atoms C121 and C221 in **MolA** and **MolB**, respectively). In addition, there is a carbonyl O atom in heterocyclic ring *C* (pivot atoms N11 and N21 in **MolA** and **MolB**, respectively), together with a potential donor, *i.e.* the –NH group on the same ring.

The Hirshfeld surface mapped over *d*_norm_ ranging from −0.329 to 1.708 for **1** show various red areas due to intra­molecular short contacts (refer to Figs. 4 ▸–7 ▸ ▸ ▸). Briefly, the analysis revealed that in **MolA** all the O atoms participate in hydrogen bonds, while only one of the nitro O atoms of ring *A* of **MolB** establishes inter­actions. A summary of these inter­actions is made in Table 3 ▸. The carbonyl O atom of heterocyclic ring *C* and the nitro atoms O129 or O229 of ring *B* are involved in hydrogen bonding between two mol­ecules with the same labels, that is A⋯A or B⋯B. These pairs inter­act in a similar way. In contrast, it seems that all the O atoms of **MolA** act as acceptors for H atoms of **MolB**. Some C⋯π inter­actions that define some substructures are identified in Table 3 ▸.

PIXEL energy calculations, as implemented in *PIXEL3.1* (Gavezzotti, 2003 ▸, 2008 ▸), give a total stabilization energy of −170.4 kJ mol^−1^ for the crystal packing, distributed as follows: *E*_Coul_ = −78.4, *E*_pol_ = −30.6, *E*_disp_ = −199.51 and *E*_rep_ = 138.2 kJ mol^−1^ for Coulombic, polarization, dispersion and repulsion energies, respectively. The polarization term is clearly less important than the Coulombic one. Partial analysis of the PIXEL calculations was also carried out to identify pairs of mol­ecules within the crystal framework that contribute most to the total energy of the packing. Fig. 8 ▸ lists the symmetry operation, the specific close contacts and the individual energy com­ponents for each mol­ecule pair. The identified mol­ecule pairs, **I** to **IX**, are depicted in Figs. 4 ▸ to 7, together with appropriate views of the Hirshfeld surface. In the figures of the mol­ecule pairs, the epimeric mol­ecules are coloured green (**MolA**) and blue (**MolB**), the partner to the specific epimer in the mol­ecular pair is coloured in standard element colours and any other relevant mol­ecule is coloured grey.

Substructures **I** and **II** connect **MolA** with **MolA** (Table 3 ▸ and Fig. 4 ▸) and subtructures **VIII** and **IX** connect **MolB** with **MolB** (Table 3 ▸ and Fig. 7 ▸). There is a similarity between substructures **I** and **VII**, as well as between substructures **II** and **IX**. Pairs **I** and **VII** are made by C_arom_—H⋯O_oxo_ inter­actions that give two isoenergetic subsets for each pair (**I**_**a**_/**I**_**b**_ and **VII**_**a**_/**VII**_**b**_). These pairs relate **MolA**⋯**MolA** and **MolB**⋯**MolB** in chains, as can be visualized in Figs. 4 ▸ and 7 ▸. The total energies for the substructures of pairs **I** and pairs **VII** differ by about 5 kJ mol^−1^ (higher value for substructure **I**) and this may be due to the presence of an additional C—H⋯π inter­action in **I** that is not detected in **VIII** **[VII?]**. The similar substructures **II_a_/II_b_** and **IX_a_/IX_b_**, are built utilizing similar C—H⋯O inter­actions, involving the O atom of the nitro group of ring *B*. Nevertheless, the total energies for those pairs also differ by about 5 kJ mol^−1^, this time with a higher value for pairs **IX** due to a higher contribution of the dispersion term.

**Figure 5:**
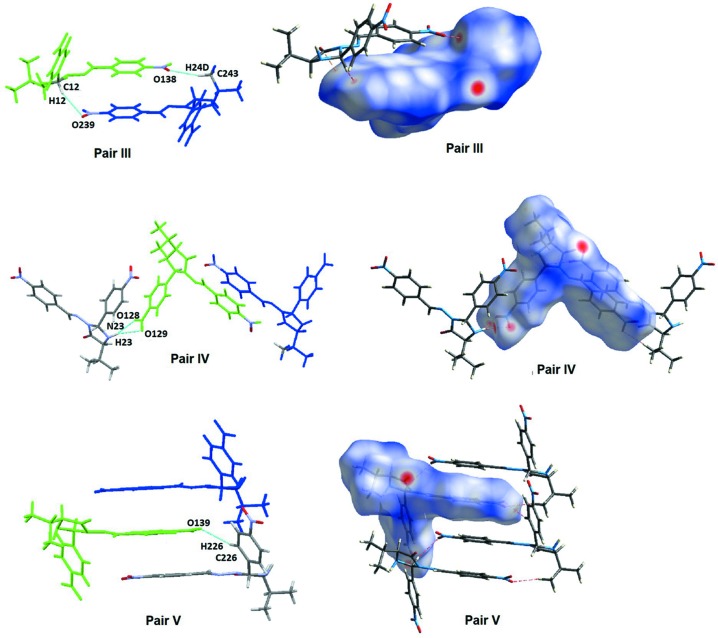
Top: inter­actions connecting mol­ecule pair III and a view of the Hirshfeld surface. Middle: inter­actions connecting mol­ecule pair IV and a view of the Hirshfeld surface. Bottom: inter­actions connecting mol­ecule pair V and a view of the Hirshfeld surface.

The mol­ecules that constitute the asymmetric unit form the nonsymmetric dimeric substructure **III**. In this substructure, the nitro O atoms of ring *A* act as acceptors in both mol­ecules, but they inter­act with different H atoms, *e.g.* (i) a methyl H atom to form the O138 ⋯H24*D*—C243 hydrogen bond in the **MolA**⋯**MolB** contact and (ii) an H atom of the imidazoline ring thereby generating an O239⋯H12—C12 hydrogen bond in the **MolB**⋯**MolA** contact (see Fig. 5 ▸).

In substructure **IV**, the N—H hydrogen of **MolB** makes a bifurcated hydrogen-bond inter­action with both O atoms of the nitro group located in ring *B* of **MolA**, *e.g.* O129⋯H23—N23 and O128⋯H23—N23 (see pair **IV** in Fig. 5 ▸). This substructure, according to the model used for the calculation of inter­actions energies, contributes the highest amount of energy to the stabilization of the crystal packing. In the substructure made by pair **V**, atom O139 of **MolA** acts as an acceptor for atom H226 of **MolB** (see Fig. 7 ▸). This layout permits a supra­molecular arrangement where aromatic rings appear to stack, but the Hirshfeld surface (HS) analysis did not reveal spots related to C⋯C close contacts that are typical of the π–π inter­actions.

**Figure 6:**
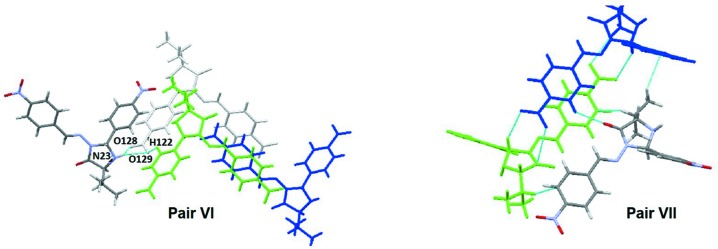
Mol­ecular pairs involved in substructures **VI** and **VII**, made by the green stick mol­ecule at (*x*, *y*, *z*) with the **[colour missing?]** colour atoms mol­ecules at (−*x*, *y* + 1, *z* + 1) (**VI**) and (*x*, *y* − 1, *z*) (**VII**). The grey mol­ecule in pair **VI** is considered to act as the conduit for electronic inter­actions, while in pair **VII**, the conduit is considered to be **MolB** (blue) of the asymmetric unit.

**Figure 7:**
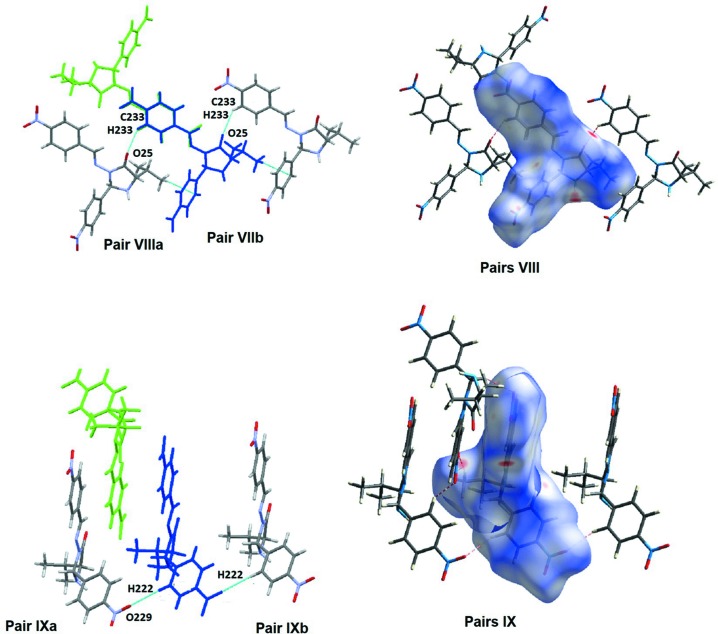
The mol­ecular pairs involved in substructures **VII** and **IX**. The figure also depicts the Hirshfeld surface images.

**Figure 8:**
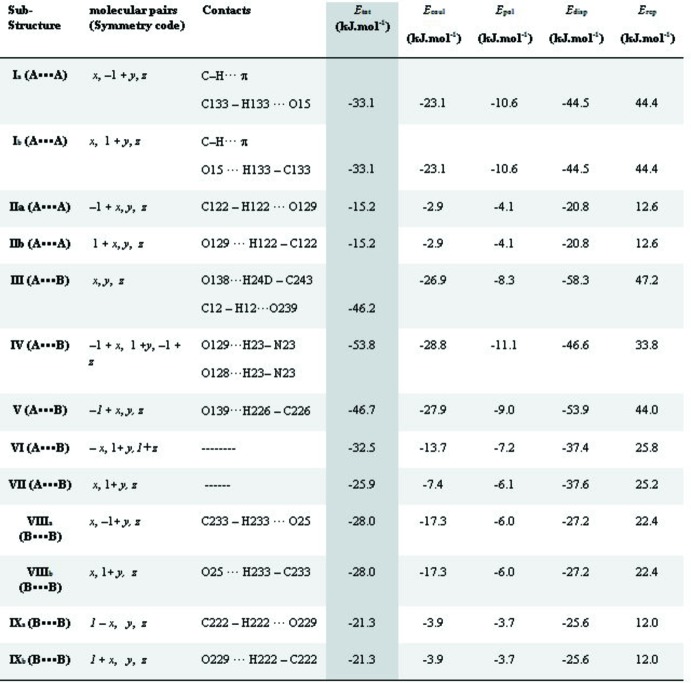
Energies, close contacts and symmetry codes of the mol­ecule pairs. A⋯A stands for **MolA**⋯**MolA** com­plexes, B⋯B for **MolB**⋯**MolB** com­plexes and A⋯B for **MolA**⋯**MolB**.

Finally, two more substructures have been identified as energetically important in the stabilization of the supra­molecular structure for **1**. Mol­ecular pairs involved in substructures **VI** and **VII**, relate the mol­ecule at (*x*, *y*, *z*) with the mol­ecules at (−*x*, *y* + 1, *z* + 1) (for **VI**) and (*x*, *y* + 1, *z*) (for **VII**). Although those mol­ecules are not connected in a classical way, the pairs make a significant contribution to the lattice stabilization energy, *i.e.* −32.5 and −25.9 kJ mol^−1^, respectively, for **VI** and **VII**. These pairs are depicted in Fig. 6 ▸, with the grey mol­ecule in pair **VI** shown in order to clarify a possible path explaining the electronic inter­actions, while in pair **VII**, the those inter­actions are made *via* **molB** of the asymmetric unit.

**Figure 9:**
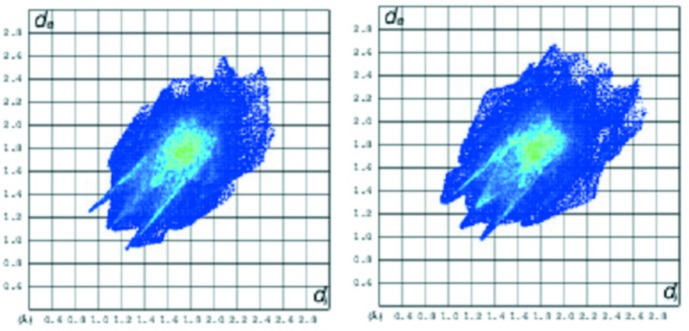
FP plots for **MolA** and **MolB**. The spikes are due to O⋯H/H⋯O contacts and the outer ones due to the N⋯H⋯N contacts.

Fig. 9 ▸ shows the fingerprint (FP) plots for **MolA** and **MolB**. The FP plots show two pairs of spikes pointing south-west and ending at (1.2; 0.9/0.9; 1.2) that are due to O⋯H/H⋯O close contacts, the light blue in the middle is due to the H⋯H and C⋯C close contacts. The percentages for atom–atom contacts were taken from the FP plots and are given in Table 4 ▸. These percentages are similar for both mol­ecules with an exception made for the O⋯H contacts that are smaller in **MolB** and the N⋯H and H⋯H contacts that are higher in **MolA.**

## Database survey   

A search of the Cambridge Structural Database (CSD, Version 5.39, August 2018 update; Groom *et al.*, 2016 ▸) was carried out. The closest structure in the database to that of **1** is the 1:1 epimeric mixture of 5-isobutyl-2-(2-nitro­phen­yl)-3-(phenyl­amino)­imidazolidin-4-one (CSD refcode VAQZUJ; Verardo *et al.*, 2003 ▸); this com­pound was also formed from a chiral reagent on reaction with a carbonyl com­pound. Other structures with a more remote relationship to **1** are 4-[(2*S*,4*S*)-4-benzyl-1-methyl-5-oxoimidazolidin-2-yl]benzo­nitrile (ZAZ­KUI; Brase *et al.*, 2012 ▸), (2*S*,5*S*)-5-benzyl-2-(4-fluoro­phen­yl)-3-methyl­imidazolidin-4-one (ZAZKOC; Brase *et al.*, 2012 ▸), 3-benzyl-5-methyl-4-oxo-2-phenyl­imidazolidin-1-ium chloride (QITMIP; Nieger, 2000 ▸), 2-*tert*-butyl-3-methyl-4-oxo-5-(penta­fluoro­benz­yl)imidazolidin-1-ium chloride (LUGTAK; Holland *et al.*, 2015 ▸), *cyclo*-[(1*S*,2*S*,3*R*,4*R*,5*R*,7*S*,10*S*,11*S*)-(*N*-{2-[(d-galactopentitol-1-yl)-4-(4-hy­droxy­benz­yl)-5-oxoimidazolin-1-yl]acet­yl}glyc­yl)-l-phenyl­alanyl-l-leucine 4′-*O*-ester (DAC­MAW; Kojic-Prodic *et al.*, 2004 ▸) and 4-[(2*S*,4*S*)-4-isopropyl-5-oxo-3-(3-oxobut­yl)-1-(pyridin-2-yl)imidazolidin-2-yl]benzo­nitrile (NURSOJ; Xu *et al.*, 2010 ▸).

## Synthesis and crystallization   

l-Valine (**2**) was converted to 2(*S*)-amino-3-methyl-1-oxo-butane­hydrazine (**3**) in two stages, as outlined in Scheme 1.

To a stirred solution of **3** (1 mmol) in ethanol (10 ml) was added 4-nitro­benzaldehyde (2.2 mmol). The reaction mixture was stirred for 20 h at 351 K and rotary evaporated. The residue was purified by column chromatography using a mixture of 9.7:0.3 (*v*/*v*) di­chloro­methane–methanol as eluent. Further purification was achieved by crystallization from ethanol. The crystal of **1** used in the structure determination was obtained by slow evaporation of an ethanol solution at room temperature.

M.p. 411–414 K. ^1^H NMR (400 MHz, DMSO-*d*_6_): δ 0.96 (6H, *m*, Me), 0.97 (6H, *m*, Me), 1.42 (2H, *m*), 2.00 (1H, *m*), 2.09 (1H, *m*), 4.06 (2H, *m*), 7.66–7.71 (4H, *m*), 7.82–7.85 (4H, *m*), 8.422–8.48 (8H, *m*).

^13^C NMR (100 MHz, DMSO-*d*_6_): δ 17.2,17.4,18.9, 29.9, 30.3, 61.8, 62.53, 124.0, 123.7, 128.1, 128.1, 128.3, 128.9, 140.2, 1140.1, 146.2, 146.3, 147.7, 148.2, 148.7, 171.1, 171.6. IR (KBr, cm^−1^): ν 3015 (*br*), 1670, 1518, 1337.

## Refinement   

Crystal data, data collection and structure refinement details are summarized in Table 5 ▸. H atoms attached to C atoms were refined as riding atoms at calculated positions. That attached to the N atom was refined.

## Supplementary Material

Crystal structure: contains datablock(s) I, global. DOI: 10.1107/S2056989019013938/lh5929sup1.cif

Structure factors: contains datablock(s) I. DOI: 10.1107/S2056989019013938/lh5929Isup2.hkl

Supporting information file. DOI: 10.1107/S2056989019013938/lh5929Isup3.cml

CCDC references: 1944779, 1944779

Additional supporting information:  crystallographic information; 3D view; checkCIF report

## Figures and Tables

**Table 1:** Hydrogen-bond geometry (Å, °)

*D*—H⋯*A*	*D*—H	H⋯*A*	*D*⋯*A*	*D*—H⋯*A*
N23—H23⋯O128^i^	0.89 (4)	2.55 (4)	3.338 (3)	147 (3)
N23—H23⋯O129^i^	0.89 (4)	2.36 (4)	3.202 (3)	159 (3)
C112—H112⋯O15	0.95	2.30	2.822 (3)	114
C212—H212⋯O25	0.95	2.16	2.832 (3)	127
C133—H133⋯O15^ii^	0.95	2.29	3.154 (3)	151
C233—H233⋯O25^iii^	0.95	2.36	3.141 (3)	139
C243—H24*D*⋯O138	0.98	2.52	3.480 (3)	165
C122—H122⋯O129^iv^	0.95	2.48	3.212 (3)	134
C222—H222⋯O229^v^	0.95	2.60	3.297 (3)	131
C226—H226⋯O139^iv^	0.95	2.57	3.197 (3)	124

Symmetry codes: (i) 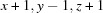; (ii) 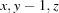; (iii) 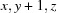; (iv) 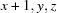; (v) 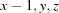.

**Table 2:** Analysis of short ring inter­actions with the *Cg*–*Cg* distances

*Cg*(*I*)	*Cg*(*J*)	*Cg*–*Cg*	Slippage
*Cg*3	*Cg*6(*x* − 1, *y*, *z*)	3.6278 (13)	1.394
*Cg*3	*Cg*6(*x*, *y*, *z*)	3.7548 (13)	1.772
*Cg*6	*Cg*3(*x* + 1, *y*, *z*)	3.6277 (13)	1.433
*Cg*6	*Cg*3(*x*, *y*, *z*)	3.7548 (13)	1.672

Notes: *Cg*(*I*) = plane number *I*, *Cg*–*Cg* = distance between ring centroids (Å), slippage = distance between *Cg*(*I*) and perpendicular projection of *Cg*(*J*) on ring *I* (Å). *Cg*3 and *Cg*6 are the centroids of the rings with pivot atoms C131 and C231, respectively.

**Table 3:** Summary of the hydrogen bonding

	*p*-NO_2_ (ring *A*)		*p*-NO_2_ (ring *B*)		(ring *C*)	
	O138/O238	O139/O239	O128/O228	O129/O229	O15/O25	N—H13/N—H23
**MolA**	A⋯B (III)	A⋯B (V)	A⋯B (IV)	A⋯B (II) A⋯B (IV)	A⋯A (I)	
**MolB**		B⋯A (III)		B⋯B (IX)	B⋯B (VIII)	B⋯A (IV)

**Table 4:** Percentages for atom–atom close contacts

1	H⋯H	H⋯O/O⋯H	H⋯C/C⋯H	C⋯C	H⋯N/N⋯H	O⋯C/C⋯O	O⋯N /N⋯O	C⋯N/N⋯C	N⋯N	O⋯O
**MolA**	36.9	35.5	11.3	4.7	2.2	3.1	1.7	1.9	1.0	1.6
**MolB**	36.5	36.2	11.5	4.7	1.6	3.3	1.7	1.9	1.0	1.6

**Table 5:** Experimental details

Crystal data	
Chemical formula	C_19_H_19_N_5_O_5_
*M*_r_	397.39
Crystal system, space group	Triclinic, *P*1
Temperature (K)	100
*a*, *b*, *c* (Å)	6.9346 (1), 8.4380 (2), 16.6963 (5)
α, β, γ (°)	79.826 (2), 89.848 (2), 80.488 (2)
*V* (Å^3^)	948.03 (4)
*Z*	2
Radiation type	Cu *K*α
μ (mm^−1^)	0.87
Crystal size (mm)	0.15 × 0.10 × 0.08

Data collection	
Diffractometer	Rigaku 007HF equipped with Varimax confocal mirrors and an C11 goniometer and HyPix 6000 detector
Absorption correction	Multi-scan (*CrysAlis PRO*; Rigaku OD, 2017 ▸)
*T*_min_, *T*_max_	0.876, 1.000
No. of measured, independent and observed [*I* > 2σ(*I*)] reflections	17290, 5885, 5634
*R*_int_	0.032
(sin θ/λ)_max_ (Å^−1^)	0.602

Refinement	
*R*[*F* ^2^ > 2σ(*F* ^2^)], *wR*(*F* ^2^), *S*	0.031, 0.089, 1.06
No. of reflections	5885
No. of parameters	535
No. of restraints	3
H-atom treatment	H atoms treated by a mixture of independent and constrained refinement
Δρ_max_, Δρ_min_ (e Å^−3^)	0.18, −0.15
Absolute structure	Flack *x* determined using 2216 quotients [(*I* ^+^) − (*I* ^−^)]/[(*I* ^+^) + (*I* ^−^)] (Parsons *et al.*, 2013 ▸)
Absolute structure parameter	0.06 (12)

Computer programs: *CrysAlis PRO* (Rigaku OD, 2017 ▸), *OSCAIL* (McArdle *et al.*, 2004 ▸), *SHELXT* (Sheldrick, 2015*a* ▸), *ShelXle* (Hübschle *et al.*, 2011 ▸), *SHELXL2017* (Sheldrick, 2015*b* ▸), *Mercury* (Macrae *et al.*, 2008 ▸), *SHELXL2017* (Sheldrick, 2015*b* ▸) and *PLATON* (Spek, 2009 ▸).

## supplementary crystallographic information

### Crystal data

**Table:**

C_19_H_19_N_5_O_5_	*Z* = 2
*M**_r_* = 397.39	*F*(000) = 416
Triclinic, *P*1	*D*_x_ = 1.392 Mg m^−^^3^
*a* = 6.9346 (1) Å	Cu *K*α radiation, λ = 1.54178 Å
*b* = 8.4380 (2) Å	Cell parameters from 10799 reflections
*c* = 16.6963 (5) Å	θ = 2.6–70.2°
α = 79.826 (2)°	µ = 0.87 mm^−^^1^
β = 89.848 (2)°	*T* = 100 K
γ = 80.488 (2)°	Block, yellow
*V* = 948.03 (4) Å^3^	0.15 × 0.10 × 0.08 mm

### Data collection

**Table:**

Rigaku 007HF equipped with Varimax confocal mirrors and an C11 goniometer and HyPix 6000 detector diffractometer	5885 independent reflections
Radiation source: Rotating anode, Rigaku 007 HF	5634 reflections with *I* > 2σ(*I*)
Varimax focusing mirrors monochromator	*R*_int_ = 0.032
Detector resolution: 10 pixels mm^-1^	θ_max_ = 68.2°, θ_min_ = 2.7°
profile data from ω–scans	*h* = −8→8
Absorption correction: multi-scan (CrysAlis PRO; Rigaku OD, 2017)	*k* = −10→10
*T*_min_ = 0.876, *T*_max_ = 1.000	*l* = −20→20
17290 measured reflections	

### Refinement

**Table:**

Refinement on *F*^2^	Hydrogen site location: mixed
Least-squares matrix: full	H atoms treated by a mixture of independent and constrained refinement
*R*[*F*^2^ > 2σ(*F*^2^)] = 0.031	*w* = 1/[σ^2^(*F*_o_^2^) + (0.0635*P*)^2^ + 0.0162*P*] where *P* = (*F*_o_^2^ + 2*F*_c_^2^)/3
*wR*(*F*^2^) = 0.089	(Δ/σ)_max_ < 0.001
*S* = 1.06	Δρ_max_ = 0.18 e Å^−^^3^
5885 reflections	Δρ_min_ = −0.15 e Å^−^^3^
535 parameters	Absolute structure: Flack *x* determined using 2216 quotients [(I+)-(I-)]/[(I+)+(I-)] (Parsons *et al.*, 2013)
3 restraints	Absolute structure parameter: 0.06 (12)

### Special details

**Table:**

Geometry. All esds (except the esd in the dihedral angle between two l.s. planes) are estimated using the full covariance matrix. The cell esds are taken into account individually in the estimation of esds in distances, angles and torsion angles; correlations between esds in cell parameters are only used when they are defined by crystal symmetry. An approximate (isotropic) treatment of cell esds is used for estimating esds involving l.s. planes.

### Fractional atomic coordinates and isotropic or equivalent isotropic displacement parameters (Å^2^)

**Table:**

	*x*	*y*	*z*	*U*_iso_*/*U*_eq_	
O15	0.4298 (3)	1.1382 (2)	0.37596 (10)	0.0355 (4)	
O25	0.7120 (3)	−0.1997 (2)	0.63494 (11)	0.0454 (5)	
O128	0.0134 (3)	0.6977 (3)	−0.07548 (12)	0.0515 (6)	
O129	−0.2170 (3)	0.7084 (3)	0.01078 (11)	0.0445 (5)	
O138	0.2174 (3)	0.1363 (3)	0.59026 (13)	0.0478 (5)	
O139	0.1723 (3)	0.2713 (3)	0.68946 (12)	0.0496 (5)	
O228	0.9876 (3)	0.2933 (3)	1.07358 (13)	0.0578 (6)	
O229	1.2157 (3)	0.2968 (3)	0.98725 (12)	0.0476 (5)	
O238	0.7903 (3)	0.8399 (3)	0.38544 (13)	0.0456 (5)	
O239	0.8329 (3)	0.6925 (3)	0.29111 (12)	0.0487 (5)	
N11	0.3637 (3)	0.9864 (2)	0.27940 (11)	0.0276 (4)	
N13	0.4764 (3)	1.1352 (3)	0.16385 (12)	0.0306 (4)	
H13	0.362 (5)	1.192 (4)	0.1427 (18)	0.034 (7)*	
N21	0.6624 (3)	−0.0012 (3)	0.71543 (12)	0.0302 (4)	
N23	0.5744 (3)	−0.1541 (3)	0.83569 (13)	0.0353 (5)	
H23	0.660 (6)	−0.187 (5)	0.877 (2)	0.058 (10)*	
N111	0.3401 (3)	0.8408 (2)	0.32899 (11)	0.0270 (4)	
N211	0.6703 (3)	0.1435 (2)	0.66255 (11)	0.0288 (4)	
C12	0.4363 (3)	0.9729 (3)	0.19724 (14)	0.0277 (5)	
H12	0.562548	0.894474	0.203010	0.033*	
C14	0.5332 (3)	1.2011 (3)	0.23511 (13)	0.0286 (5)	
H14	0.678098	1.168896	0.243608	0.034*	
C15	0.4376 (3)	1.1108 (3)	0.30696 (13)	0.0287 (5)	
C22	0.5853 (4)	0.0145 (3)	0.79628 (14)	0.0298 (5)	
H22	0.450305	0.079596	0.789649	0.036*	
C24	0.6057 (4)	−0.2618 (3)	0.77444 (14)	0.0335 (5)	
H24	0.715645	−0.353217	0.793820	0.040*	
C25	0.6675 (4)	−0.1559 (3)	0.69896 (15)	0.0348 (5)	
C112	0.2670 (3)	0.8488 (3)	0.39906 (14)	0.0279 (5)	
H112	0.225845	0.951561	0.414946	0.033*	
C121	0.2980 (3)	0.9158 (3)	0.14341 (14)	0.0262 (5)	
C122	0.3699 (4)	0.8750 (3)	0.07013 (14)	0.0308 (5)	
H122	0.499729	0.887882	0.055715	0.037*	
C123	0.2556 (4)	0.8164 (3)	0.01828 (14)	0.0320 (5)	
H123	0.304016	0.790033	−0.031853	0.038*	
C124	0.0687 (4)	0.7973 (3)	0.04175 (14)	0.0293 (5)	
C125	−0.0089 (3)	0.8388 (3)	0.11285 (14)	0.0312 (5)	
H125	−0.138837	0.825399	0.126857	0.037*	
C126	0.1067 (4)	0.9007 (3)	0.16358 (14)	0.0313 (5)	
H126	0.054796	0.932735	0.212100	0.038*	
N127	−0.0521 (3)	0.7287 (3)	−0.01117 (12)	0.0323 (5)	
C131	0.2478 (3)	0.6970 (3)	0.45425 (13)	0.0261 (5)	
C132	0.2849 (3)	0.5462 (3)	0.42896 (14)	0.0264 (5)	
H132	0.320914	0.541502	0.374383	0.032*	
C133	0.2700 (3)	0.4040 (3)	0.48178 (14)	0.0287 (5)	
H133	0.295345	0.301262	0.464512	0.034*	
C134	0.2168 (3)	0.4152 (3)	0.56112 (14)	0.0302 (5)	
C135	0.1772 (3)	0.5612 (3)	0.58830 (14)	0.0318 (5)	
H135	0.140140	0.564508	0.642882	0.038*	
C136	0.1922 (3)	0.7036 (3)	0.53455 (14)	0.0307 (5)	
H136	0.164724	0.805932	0.552131	0.037*	
N137	0.2007 (3)	0.2631 (3)	0.61757 (13)	0.0378 (5)	
C141	0.4849 (4)	1.3855 (3)	0.22729 (14)	0.0324 (5)	
H141	0.522467	1.411918	0.280582	0.039*	
C142	0.6066 (5)	1.4719 (4)	0.16285 (18)	0.0451 (7)	
H14D	0.580595	1.589617	0.163030	0.068*	
H14E	0.571639	1.451708	0.109156	0.068*	
H14F	0.745792	1.430079	0.174926	0.068*	
C143	0.2666 (4)	1.4489 (3)	0.21372 (16)	0.0387 (6)	
H14A	0.238790	1.563576	0.220178	0.058*	
H14B	0.193577	1.384089	0.253620	0.058*	
H14C	0.227059	1.439805	0.158629	0.058*	
C212	0.7367 (3)	0.1361 (3)	0.59108 (15)	0.0301 (5)	
H212	0.777043	0.033090	0.575349	0.036*	
C221	0.7088 (3)	0.0917 (3)	0.84770 (14)	0.0282 (5)	
C222	0.6347 (4)	0.1260 (3)	0.92174 (14)	0.0299 (5)	
H222	0.506428	0.107886	0.936016	0.036*	
C223	0.7456 (4)	0.1858 (3)	0.97467 (14)	0.0308 (5)	
H223	0.696374	0.206566	1.025638	0.037*	
C224	0.9301 (4)	0.2146 (3)	0.95128 (14)	0.0286 (5)	
C225	1.0058 (4)	0.1867 (3)	0.87724 (15)	0.0325 (5)	
H225	1.131517	0.210277	0.862046	0.039*	
C226	0.8942 (3)	0.1235 (3)	0.82574 (14)	0.0301 (5)	
H226	0.944672	0.101824	0.775113	0.036*	
N227	1.0519 (3)	0.2745 (3)	1.00771 (12)	0.0339 (5)	
C231	0.7499 (3)	0.2873 (3)	0.53383 (13)	0.0277 (5)	
C232	0.7186 (3)	0.4406 (3)	0.55683 (14)	0.0287 (5)	
H232	0.685933	0.449496	0.611332	0.034*	
C233	0.7347 (3)	0.5795 (3)	0.50108 (15)	0.0299 (5)	
H233	0.712742	0.683984	0.516467	0.036*	
C234	0.7840 (3)	0.5624 (3)	0.42183 (15)	0.0307 (5)	
C235	0.8170 (3)	0.4122 (3)	0.39713 (14)	0.0314 (5)	
H235	0.850887	0.403867	0.342684	0.038*	
C236	0.7993 (3)	0.2746 (3)	0.45354 (14)	0.0303 (5)	
H236	0.820955	0.170474	0.437723	0.036*	
N237	0.8038 (3)	0.7084 (3)	0.36213 (13)	0.0366 (5)	
C241	0.4241 (4)	−0.3336 (3)	0.75728 (15)	0.0345 (5)	
H241	0.445003	−0.378380	0.705741	0.041*	
C242	0.3982 (5)	−0.4738 (4)	0.82522 (19)	0.0455 (7)	
H24A	0.515569	−0.557757	0.830591	0.068*	
H24B	0.284532	−0.520913	0.812321	0.068*	
H24C	0.377662	−0.432928	0.876535	0.068*	
C243	0.2417 (4)	−0.2027 (3)	0.74492 (16)	0.0369 (5)	
H24D	0.259555	−0.117069	0.698937	0.055*	
H24E	0.219738	−0.155144	0.794304	0.055*	
H24F	0.128424	−0.251832	0.733575	0.055*	

### Atomic displacement parameters (Å^2^)

**Table:**

	*U*^11^	*U*^22^	*U*^33^	*U*^12^	*U*^13^	*U*^23^
O15	0.0480 (10)	0.0339 (9)	0.0271 (8)	−0.0136 (8)	−0.0009 (7)	−0.0057 (7)
O25	0.0636 (12)	0.0408 (11)	0.0385 (10)	−0.0199 (9)	0.0130 (9)	−0.0150 (8)
O128	0.0459 (11)	0.0821 (16)	0.0359 (11)	−0.0172 (10)	0.0023 (8)	−0.0293 (11)
O129	0.0418 (10)	0.0597 (13)	0.0372 (10)	−0.0232 (9)	0.0006 (8)	−0.0087 (9)
O138	0.0493 (12)	0.0346 (11)	0.0559 (12)	−0.0119 (9)	−0.0051 (9)	0.0066 (9)
O139	0.0474 (11)	0.0619 (13)	0.0327 (10)	−0.0116 (10)	0.0032 (8)	0.0120 (9)
O228	0.0529 (12)	0.0899 (18)	0.0419 (11)	−0.0211 (12)	0.0018 (9)	−0.0340 (12)
O229	0.0443 (11)	0.0625 (14)	0.0433 (11)	−0.0252 (10)	−0.0014 (9)	−0.0134 (10)
O238	0.0378 (10)	0.0405 (11)	0.0533 (12)	−0.0083 (8)	−0.0046 (8)	0.0074 (9)
O239	0.0458 (11)	0.0592 (13)	0.0333 (10)	−0.0063 (9)	0.0048 (8)	0.0101 (9)
N11	0.0356 (10)	0.0262 (11)	0.0222 (9)	−0.0108 (8)	0.0005 (8)	−0.0023 (8)
N13	0.0390 (11)	0.0278 (11)	0.0261 (10)	−0.0118 (9)	−0.0003 (8)	−0.0016 (8)
N21	0.0349 (11)	0.0332 (12)	0.0243 (9)	−0.0125 (9)	0.0008 (8)	−0.0039 (8)
N23	0.0469 (12)	0.0353 (12)	0.0261 (10)	−0.0177 (10)	−0.0048 (9)	−0.0021 (9)
N111	0.0300 (10)	0.0274 (11)	0.0238 (10)	−0.0099 (8)	−0.0039 (7)	−0.0002 (8)
N211	0.0295 (10)	0.0339 (11)	0.0237 (10)	−0.0116 (9)	−0.0026 (8)	−0.0013 (8)
C12	0.0321 (12)	0.0283 (13)	0.0228 (11)	−0.0074 (10)	0.0006 (9)	−0.0025 (9)
C14	0.0310 (11)	0.0278 (12)	0.0274 (11)	−0.0077 (9)	−0.0021 (9)	−0.0032 (9)
C15	0.0329 (12)	0.0258 (12)	0.0267 (11)	−0.0052 (9)	−0.0034 (9)	−0.0025 (9)
C22	0.0325 (12)	0.0348 (13)	0.0226 (11)	−0.0112 (10)	0.0019 (9)	−0.0012 (10)
C24	0.0371 (13)	0.0312 (13)	0.0314 (12)	−0.0073 (10)	−0.0026 (10)	−0.0018 (9)
C25	0.0360 (12)	0.0358 (14)	0.0342 (12)	−0.0109 (10)	0.0010 (10)	−0.0060 (10)
C112	0.0275 (11)	0.0307 (12)	0.0265 (11)	−0.0080 (9)	−0.0012 (9)	−0.0047 (9)
C121	0.0338 (12)	0.0202 (11)	0.0231 (11)	−0.0057 (9)	−0.0006 (9)	0.0010 (8)
C122	0.0350 (12)	0.0326 (13)	0.0253 (12)	−0.0106 (10)	0.0034 (9)	−0.0022 (10)
C123	0.0394 (13)	0.0325 (13)	0.0247 (12)	−0.0093 (10)	0.0031 (10)	−0.0036 (9)
C124	0.0362 (12)	0.0261 (12)	0.0244 (11)	−0.0064 (10)	−0.0044 (9)	−0.0003 (9)
C125	0.0295 (12)	0.0357 (13)	0.0273 (12)	−0.0064 (10)	0.0001 (9)	−0.0022 (10)
C126	0.0352 (12)	0.0343 (13)	0.0238 (11)	−0.0045 (10)	−0.0002 (9)	−0.0045 (9)
N127	0.0360 (11)	0.0325 (11)	0.0286 (11)	−0.0083 (9)	−0.0026 (8)	−0.0030 (8)
C131	0.0226 (11)	0.0341 (13)	0.0226 (11)	−0.0088 (10)	−0.0028 (8)	−0.0037 (9)
C132	0.0254 (11)	0.0341 (13)	0.0209 (10)	−0.0095 (9)	−0.0005 (8)	−0.0043 (9)
C133	0.0229 (11)	0.0337 (13)	0.0306 (12)	−0.0081 (10)	−0.0035 (9)	−0.0058 (10)
C134	0.0227 (11)	0.0378 (14)	0.0278 (11)	−0.0097 (10)	−0.0037 (9)	0.0047 (10)
C135	0.0277 (11)	0.0458 (16)	0.0228 (11)	−0.0110 (11)	−0.0006 (9)	−0.0038 (10)
C136	0.0307 (12)	0.0378 (14)	0.0256 (11)	−0.0095 (10)	0.0002 (9)	−0.0078 (10)
N137	0.0258 (10)	0.0467 (15)	0.0366 (12)	−0.0093 (10)	−0.0032 (9)	0.0067 (10)
C141	0.0403 (13)	0.0290 (12)	0.0290 (11)	−0.0094 (10)	−0.0022 (9)	−0.0046 (9)
C142	0.0623 (18)	0.0298 (14)	0.0442 (15)	−0.0159 (13)	0.0078 (13)	−0.0020 (12)
C143	0.0437 (14)	0.0309 (13)	0.0402 (13)	−0.0031 (11)	−0.0100 (11)	−0.0058 (10)
C212	0.0301 (11)	0.0353 (13)	0.0274 (12)	−0.0114 (10)	−0.0006 (9)	−0.0071 (10)
C221	0.0336 (12)	0.0271 (12)	0.0233 (11)	−0.0074 (10)	−0.0015 (9)	−0.0008 (9)
C222	0.0325 (12)	0.0309 (13)	0.0260 (12)	−0.0089 (10)	0.0016 (9)	−0.0009 (9)
C223	0.0375 (13)	0.0326 (13)	0.0221 (11)	−0.0064 (10)	0.0014 (9)	−0.0034 (9)
C224	0.0374 (12)	0.0234 (11)	0.0245 (11)	−0.0074 (10)	−0.0044 (9)	−0.0012 (9)
C225	0.0333 (12)	0.0359 (13)	0.0296 (12)	−0.0111 (10)	0.0015 (10)	−0.0045 (10)
C226	0.0323 (12)	0.0347 (13)	0.0242 (11)	−0.0098 (10)	0.0025 (9)	−0.0038 (9)
N227	0.0400 (12)	0.0325 (12)	0.0293 (11)	−0.0056 (9)	−0.0053 (9)	−0.0058 (9)
C231	0.0224 (11)	0.0391 (14)	0.0230 (11)	−0.0099 (10)	−0.0018 (9)	−0.0049 (10)
C232	0.0235 (11)	0.0407 (14)	0.0236 (11)	−0.0088 (10)	−0.0039 (9)	−0.0067 (10)
C233	0.0234 (11)	0.0355 (14)	0.0314 (12)	−0.0081 (10)	−0.0036 (9)	−0.0048 (10)
C234	0.0223 (11)	0.0414 (14)	0.0267 (11)	−0.0086 (10)	−0.0037 (9)	0.0015 (10)
C235	0.0254 (11)	0.0476 (15)	0.0216 (11)	−0.0098 (10)	−0.0011 (9)	−0.0038 (10)
C236	0.0266 (11)	0.0406 (14)	0.0257 (11)	−0.0110 (10)	−0.0008 (9)	−0.0068 (10)
N237	0.0253 (10)	0.0434 (14)	0.0364 (12)	−0.0071 (9)	−0.0013 (8)	0.0070 (10)
C241	0.0442 (14)	0.0300 (13)	0.0316 (12)	−0.0130 (11)	0.0002 (10)	−0.0051 (10)
C242	0.0566 (17)	0.0313 (15)	0.0480 (16)	−0.0143 (13)	0.0027 (13)	0.0013 (12)
C243	0.0381 (13)	0.0378 (14)	0.0362 (12)	−0.0109 (11)	−0.0026 (10)	−0.0061 (10)

### Geometric parameters (Å, º)

**Table:**

O15—C15	1.213 (3)	C132—H132	0.9500
O25—C25	1.214 (3)	C133—C134	1.389 (3)
O128—N127	1.219 (3)	C133—H133	0.9500
O129—N127	1.229 (3)	C134—C135	1.373 (4)
O138—N137	1.224 (3)	C134—N137	1.472 (3)
O139—N137	1.228 (3)	C135—C136	1.386 (4)
O228—N227	1.212 (3)	C135—H135	0.9500
O229—N227	1.220 (3)	C136—H136	0.9500
O238—N237	1.229 (3)	C141—C143	1.523 (4)
O239—N237	1.229 (3)	C141—C142	1.524 (4)
N11—C15	1.388 (3)	C141—H141	1.0000
N11—N111	1.388 (3)	C142—H14D	0.9800
N11—C12	1.477 (3)	C142—H14E	0.9800
N13—C12	1.456 (3)	C142—H14F	0.9800
N13—C14	1.479 (3)	C143—H14A	0.9800
N13—H13	0.89 (3)	C143—H14B	0.9800
N21—C25	1.375 (3)	C143—H14C	0.9800
N21—N211	1.384 (3)	C212—C231	1.469 (3)
N21—C22	1.470 (3)	C212—H212	0.9500
N23—C22	1.471 (3)	C221—C226	1.391 (3)
N23—C24	1.477 (3)	C221—C222	1.396 (3)
N23—H23	0.89 (4)	C222—C223	1.385 (3)
N111—C112	1.282 (3)	C222—H222	0.9500
N211—C212	1.286 (3)	C223—C224	1.384 (3)
C12—C121	1.507 (3)	C223—H223	0.9500
C12—H12	1.0000	C224—C225	1.385 (3)
C14—C15	1.516 (3)	C224—N227	1.473 (3)
C14—C141	1.518 (3)	C225—C226	1.387 (3)
C14—H14	1.0000	C225—H225	0.9500
C22—C221	1.510 (3)	C226—H226	0.9500
C22—H22	1.0000	C231—C232	1.396 (4)
C24—C25	1.515 (3)	C231—C236	1.401 (3)
C24—C241	1.533 (3)	C232—C233	1.382 (4)
C24—H24	1.0000	C232—H232	0.9500
C112—C131	1.465 (3)	C233—C234	1.392 (3)
C112—H112	0.9500	C233—H233	0.9500
C121—C126	1.389 (3)	C234—C235	1.385 (4)
C121—C122	1.397 (3)	C234—N237	1.467 (3)
C122—C123	1.381 (3)	C235—C236	1.382 (4)
C122—H122	0.9500	C235—H235	0.9500
C123—C124	1.379 (4)	C236—H236	0.9500
C123—H123	0.9500	C241—C242	1.521 (4)
C124—C125	1.379 (3)	C241—C243	1.524 (4)
C124—N127	1.471 (3)	C241—H241	1.0000
C125—C126	1.389 (3)	C242—H24A	0.9800
C125—H125	0.9500	C242—H24B	0.9800
C126—H126	0.9500	C242—H24C	0.9800
C131—C132	1.395 (3)	C243—H24D	0.9800
C131—C136	1.402 (3)	C243—H24E	0.9800
C132—C133	1.377 (3)	C243—H24F	0.9800
			
C15—N11—N111	123.74 (18)	C131—C136—H136	119.9
C15—N11—C12	109.37 (18)	O138—N137—O139	123.9 (2)
N111—N11—C12	115.87 (18)	O138—N137—C134	118.2 (2)
C12—N13—C14	104.96 (18)	O139—N137—C134	117.8 (2)
C12—N13—H13	105.6 (19)	C14—C141—C143	112.28 (19)
C14—N13—H13	109.4 (19)	C14—C141—C142	111.7 (2)
C25—N21—N211	129.5 (2)	C143—C141—C142	112.2 (2)
C25—N21—C22	112.2 (2)	C14—C141—H141	106.7
N211—N21—C22	116.06 (19)	C143—C141—H141	106.7
C22—N23—C24	109.21 (19)	C142—C141—H141	106.7
C22—N23—H23	111 (2)	C141—C142—H14D	109.5
C24—N23—H23	112 (2)	C141—C142—H14E	109.5
C112—N111—N11	117.58 (19)	H14D—C142—H14E	109.5
C212—N211—N21	118.4 (2)	C141—C142—H14F	109.5
N13—C12—N11	104.41 (18)	H14D—C142—H14F	109.5
N13—C12—C121	112.35 (19)	H14E—C142—H14F	109.5
N11—C12—C121	114.42 (19)	C141—C143—H14A	109.5
N13—C12—H12	108.5	C141—C143—H14B	109.5
N11—C12—H12	108.5	H14A—C143—H14B	109.5
C121—C12—H12	108.5	C141—C143—H14C	109.5
N13—C14—C15	105.22 (17)	H14A—C143—H14C	109.5
N13—C14—C141	115.33 (19)	H14B—C143—H14C	109.5
C15—C14—C141	113.03 (19)	N211—C212—C231	119.8 (2)
N13—C14—H14	107.6	N211—C212—H212	120.1
C15—C14—H14	107.6	C231—C212—H212	120.1
C141—C14—H14	107.6	C226—C221—C222	119.4 (2)
O15—C15—N11	125.9 (2)	C226—C221—C22	122.8 (2)
O15—C15—C14	127.3 (2)	C222—C221—C22	117.8 (2)
N11—C15—C14	106.73 (18)	C223—C222—C221	121.0 (2)
N21—C22—N23	104.4 (2)	C223—C222—H222	119.5
N21—C22—C221	114.65 (19)	C221—C222—H222	119.5
N23—C22—C221	110.4 (2)	C224—C223—C222	118.1 (2)
N21—C22—H22	109.1	C224—C223—H223	120.9
N23—C22—H22	109.1	C222—C223—H223	120.9
C221—C22—H22	109.1	C223—C224—C225	122.4 (2)
N23—C24—C25	104.9 (2)	C223—C224—N227	118.9 (2)
N23—C24—C241	113.3 (2)	C225—C224—N227	118.7 (2)
C25—C24—C241	111.6 (2)	C224—C225—C226	118.6 (2)
N23—C24—H24	108.9	C224—C225—H225	120.7
C25—C24—H24	108.9	C226—C225—H225	120.7
C241—C24—H24	108.9	C225—C226—C221	120.5 (2)
O25—C25—N21	126.1 (2)	C225—C226—H226	119.8
O25—C25—C24	125.9 (2)	C221—C226—H226	119.8
N21—C25—C24	108.0 (2)	O228—N227—O229	123.0 (2)
N111—C112—C131	119.0 (2)	O228—N227—C224	118.5 (2)
N111—C112—H112	120.5	O229—N227—C224	118.5 (2)
C131—C112—H112	120.5	C232—C231—C236	119.5 (2)
C126—C121—C122	119.4 (2)	C232—C231—C212	122.6 (2)
C126—C121—C12	123.4 (2)	C236—C231—C212	117.9 (2)
C122—C121—C12	117.2 (2)	C233—C232—C231	120.6 (2)
C123—C122—C121	121.2 (2)	C233—C232—H232	119.7
C123—C122—H122	119.4	C231—C232—H232	119.7
C121—C122—H122	119.4	C232—C233—C234	118.4 (2)
C124—C123—C122	117.7 (2)	C232—C233—H233	120.8
C124—C123—H123	121.1	C234—C233—H233	120.8
C122—C123—H123	121.1	C235—C234—C233	122.5 (2)
C125—C124—C123	122.8 (2)	C235—C234—N237	118.5 (2)
C125—C124—N127	118.7 (2)	C233—C234—N237	119.0 (2)
C123—C124—N127	118.4 (2)	C236—C235—C234	118.4 (2)
C124—C125—C126	118.7 (2)	C236—C235—H235	120.8
C124—C125—H125	120.7	C234—C235—H235	120.8
C126—C125—H125	120.7	C235—C236—C231	120.6 (2)
C121—C126—C125	120.1 (2)	C235—C236—H236	119.7
C121—C126—H126	120.0	C231—C236—H236	119.7
C125—C126—H126	120.0	O239—N237—O238	123.4 (2)
O128—N127—O129	122.8 (2)	O239—N237—C234	118.0 (2)
O128—N127—C124	118.8 (2)	O238—N237—C234	118.6 (2)
O129—N127—C124	118.3 (2)	C242—C241—C243	111.2 (2)
C132—C131—C136	119.3 (2)	C242—C241—C24	110.5 (2)
C132—C131—C112	121.6 (2)	C243—C241—C24	111.3 (2)
C136—C131—C112	119.1 (2)	C242—C241—H241	107.9
C133—C132—C131	121.1 (2)	C243—C241—H241	107.9
C133—C132—H132	119.5	C24—C241—H241	107.9
C131—C132—H132	119.5	C241—C242—H24A	109.5
C132—C133—C134	118.0 (2)	C241—C242—H24B	109.5
C132—C133—H133	121.0	H24A—C242—H24B	109.5
C134—C133—H133	121.0	C241—C242—H24C	109.5
C135—C134—C133	122.9 (2)	H24A—C242—H24C	109.5
C135—C134—N137	119.2 (2)	H24B—C242—H24C	109.5
C133—C134—N137	118.0 (2)	C241—C243—H24D	109.5
C134—C135—C136	118.7 (2)	C241—C243—H24E	109.5
C134—C135—H135	120.7	H24D—C243—H24E	109.5
C136—C135—H135	120.7	C241—C243—H24F	109.5
C135—C136—C131	120.1 (2)	H24D—C243—H24F	109.5
C135—C136—H136	119.9	H24E—C243—H24F	109.5
			
C15—N11—N111—C112	43.7 (3)	C136—C131—C132—C133	−0.8 (3)
C12—N11—N111—C112	−176.19 (19)	C112—C131—C132—C133	178.75 (19)
C25—N21—N211—C212	−18.6 (3)	C131—C132—C133—C134	0.0 (3)
C22—N21—N211—C212	179.98 (19)	C132—C133—C134—C135	0.6 (3)
C14—N13—C12—N11	30.7 (2)	C132—C133—C134—N137	179.92 (18)
C14—N13—C12—C121	155.3 (2)	C133—C134—C135—C136	−0.5 (3)
C15—N11—C12—N13	−25.8 (2)	N137—C134—C135—C136	−179.8 (2)
N111—N11—C12—N13	−171.33 (18)	C134—C135—C136—C131	−0.3 (3)
C15—N11—C12—C121	−149.0 (2)	C132—C131—C136—C135	0.9 (3)
N111—N11—C12—C121	65.4 (3)	C112—C131—C136—C135	−178.6 (2)
C12—N13—C14—C15	−25.1 (2)	C135—C134—N137—O138	172.8 (2)
C12—N13—C14—C141	−150.3 (2)	C133—C134—N137—O138	−6.5 (3)
N111—N11—C15—O15	−26.3 (4)	C135—C134—N137—O139	−7.6 (3)
C12—N11—C15—O15	−168.6 (2)	C133—C134—N137—O139	173.1 (2)
N111—N11—C15—C14	152.1 (2)	N13—C14—C141—C143	59.6 (3)
C12—N11—C15—C14	9.9 (3)	C15—C14—C141—C143	−61.5 (3)
N13—C14—C15—O15	−172.1 (2)	N13—C14—C141—C142	−67.5 (3)
C141—C14—C15—O15	−45.4 (3)	C15—C14—C141—C142	171.4 (2)
N13—C14—C15—N11	9.5 (2)	N21—N211—C212—C231	−179.07 (18)
C141—C14—C15—N11	136.1 (2)	N21—C22—C221—C226	−9.1 (3)
C25—N21—C22—N23	9.8 (3)	N23—C22—C221—C226	108.5 (3)
N211—N21—C22—N23	174.33 (17)	N21—C22—C221—C222	173.6 (2)
C25—N21—C22—C221	130.7 (2)	N23—C22—C221—C222	−68.8 (3)
N211—N21—C22—C221	−64.7 (3)	C226—C221—C222—C223	−2.2 (4)
C24—N23—C22—N21	−11.1 (3)	C22—C221—C222—C223	175.3 (2)
C24—N23—C22—C221	−134.8 (2)	C221—C222—C223—C224	1.5 (4)
C22—N23—C24—C25	8.6 (3)	C222—C223—C224—C225	0.4 (4)
C22—N23—C24—C241	−113.4 (2)	C222—C223—C224—N227	−178.5 (2)
N211—N21—C25—O25	12.8 (4)	C223—C224—C225—C226	−1.7 (4)
C22—N21—C25—O25	174.8 (2)	N227—C224—C225—C226	177.2 (2)
N211—N21—C25—C24	−166.5 (2)	C224—C225—C226—C221	1.1 (4)
C22—N21—C25—C24	−4.6 (3)	C222—C221—C226—C225	0.8 (4)
N23—C24—C25—O25	178.1 (2)	C22—C221—C226—C225	−176.5 (2)
C241—C24—C25—O25	−58.8 (3)	C223—C224—N227—O228	1.5 (3)
N23—C24—C25—N21	−2.5 (3)	C225—C224—N227—O228	−177.5 (2)
C241—C24—C25—N21	120.6 (2)	C223—C224—N227—O229	178.7 (2)
N11—N111—C112—C131	−177.45 (18)	C225—C224—N227—O229	−0.3 (3)
N13—C12—C121—C126	−109.4 (3)	N211—C212—C231—C232	9.0 (3)
N11—C12—C121—C126	9.5 (3)	N211—C212—C231—C236	−172.1 (2)
N13—C12—C121—C122	71.3 (3)	C236—C231—C232—C233	0.4 (3)
N11—C12—C121—C122	−169.9 (2)	C212—C231—C232—C233	179.3 (2)
C126—C121—C122—C123	−1.5 (4)	C231—C232—C233—C234	−0.4 (3)
C12—C121—C122—C123	177.8 (2)	C232—C233—C234—C235	0.1 (3)
C121—C122—C123—C124	−0.8 (4)	C232—C233—C234—N237	−179.35 (19)
C122—C123—C124—C125	2.0 (4)	C233—C234—C235—C236	0.2 (3)
C122—C123—C124—N127	−177.5 (2)	N237—C234—C235—C236	179.65 (19)
C123—C124—C125—C126	−0.8 (4)	C234—C235—C236—C231	−0.2 (3)
N127—C124—C125—C126	178.8 (2)	C232—C231—C236—C235	−0.1 (3)
C122—C121—C126—C125	2.8 (4)	C212—C231—C236—C235	−179.0 (2)
C12—C121—C126—C125	−176.5 (2)	C235—C234—N237—O239	5.9 (3)
C124—C125—C126—C121	−1.7 (4)	C233—C234—N237—O239	−174.7 (2)
C125—C124—N127—O128	177.3 (2)	C235—C234—N237—O238	−174.1 (2)
C123—C124—N127—O128	−3.1 (4)	C233—C234—N237—O238	5.3 (3)
C125—C124—N127—O129	−1.2 (4)	N23—C24—C241—C242	−77.5 (3)
C123—C124—N127—O129	178.4 (2)	C25—C24—C241—C242	164.3 (2)
N111—C112—C131—C132	−7.8 (3)	N23—C24—C241—C243	46.6 (3)
N111—C112—C131—C136	171.7 (2)	C25—C24—C241—C243	−71.7 (3)

### Hydrogen-bond geometry (Å, º)

**Table:**

*D*—H···*A*	*D*—H	H···*A*	*D*···*A*	*D*—H···*A*
N23—H23···O128^i^	0.89 (4)	2.55 (4)	3.338 (3)	147 (3)
N23—H23···O129^i^	0.89 (4)	2.36 (4)	3.202 (3)	159 (3)
C112—H112···O15	0.95	2.30	2.822 (3)	114
C212—H212···O25	0.95	2.16	2.832 (3)	127
C133—H133···O15^ii^	0.95	2.29	3.154 (3)	151
C233—H233···O25^iii^	0.95	2.36	3.141 (3)	139
C243—H24*D*···O138	0.98	2.52	3.480 (3)	165
C122—H122···O129^iv^	0.95	2.48	3.212 (3)	134
C222—H222···O229^v^	0.95	2.60	3.297 (3)	131
C226—H226···O139^iv^	0.95	2.57	3.197 (3)	124

Symmetry codes: (i) *x*+1, *y*−1, *z*+1; (ii) *x*, *y*−1, *z*; (iii) *x*, *y*+1, *z*; (iv) *x*+1, *y*, *z*; (v) *x*−1, *y*, *z*.
